# Recruitment of Mobile Genetic Elements for Diverse Cellular Functions in Prokaryotes

**DOI:** 10.3389/fmolb.2022.821197

**Published:** 2022-03-24

**Authors:** Sean Benler, Eugene V. Koonin

**Affiliations:** National Center for Biotechnology Information, National Library of Medicine, National Institutes of Health, Bethesda, MD, United States

**Keywords:** mobile genetic element, exaptation, antivirus defense mechanisms, biological conflict systems, horizontal gene transfer

## Abstract

Prokaryotic genomes are replete with mobile genetic elements (MGE) that span a continuum of replication autonomy. On numerous occasions during microbial evolution, diverse MGE lose their autonomy altogether but, rather than being quickly purged from the host genome, assume a new function that benefits the host, rendering the immobilized MGE subject to purifying selection, and resulting in its vertical inheritance. This mini-review highlights the diversity of the repurposed (exapted) MGE as well as the plethora of cellular functions that they perform. The principal contribution of the exaptation of MGE and their components is to the prokaryotic functional systems involved in biological conflicts, and in particular, defense against viruses and other MGE. This evolutionary entanglement between MGE and defense systems appears to stem both from mechanistic similarities and from similar evolutionary predicaments whereby both MGEs and defense systems tend to incur fitness costs to the hosts and thereby evolve mechanisms for survival including horizontal mobility, causing host addiction, and exaptation for functions beneficial to the host. The examples discussed demonstrate that the identity of an MGE, overall mobility and relationship with the host cell (mutualistic, symbiotic, commensal, or parasitic) are all factors that affect exaptation.

## Introduction

Coevolution of mobile genetic elements (MGEs) and cellular organisms spans billions of years and is thought to have spurred innumerable evolutionary innovations (Werren, 2011; Koonin, 2016). Diverse MGEs that jointly comprise a vast mobilome are associated with essentially all known cellular organisms, with the possible exception of some intracellular parasitic and symbiotic bacteria (Frost et al., 2005; Iranzo et al., 2016; Carr et al., 2021). The relationship between MGEs and their cellular hosts spans a continuum, ranging from mutualistic to parasitic, changing between different MGE-host pairs as well as within the same pair over time (Jalasvuori and Koonin, 2015). One form of this dynamic relationship can be described as antagonistic coevolution, where an incessant arms-race takes place between the MGE and the host, driving the evolution of ornate defense and counter-defense systems encoded by both parties (Duggal and Emerman, 2012; Feschotte and Gilbert, 2012; Bernheim and Sorek, 2020). Intensive investigation of defense and counter-defense systems, such as CRISPR-Cas, uncovered back-and-forth shuttling of the system’s components between MGEs and their hosts over evolutionary time (Koonin et al., 2019). The evolutionary entanglement between MGEs and hosts is not restricted to defense systems alone, but to our knowledge, the full extent of such exchanges has not been systematically reviewed.

One key aspect of the evolutionary exchange between MGEs and cells is exaptation. Exaptation refers to the recruitment, driven by natural selection, of a biological entity for a new role unrelated to the original one with respect to the biological function, while exploiting the mechanistic features of the recruited entity (Gould and Vrba, 1982; Gould, 1997). We use this term instead of the more common “domestication” to emphasize the functional shifts that occur upon recruitment of MGEs or their components for cellular functions (Koonin and Krupovic, 2018; Koonin and Makarova, 2022). For example, a nuclease originally involved in transposition of a distinct variety of IS200/IS605-like transposons evolved into Cas9, the effector of type II CRISPR-Cas adaptive immunity (Kapitonov et al., 2016; Altae-Tran et al., 2021), whereas the transposase of another type of transposons gave rise to Cas1, the integrase involved in spacer acquisition by CRISPR-Cas systems (Krupovic et al., 2014). Exaptation can occur at different “depths”, ranging from the recruitment of an entire MGE for a new role to repurposing of a single component of an MGE (Koonin and Krupovic, 2018). Exaptation of MGE genes is linked to horizontal gene transfer (HGT), the major evolutionary force in prokaryotes (Doolittle, 1999; Koonin et al., 2001). Due to their high horizontal mobility, MGE double as vehicles for HGT. Indeed, some MGE carry large repertoires of diverse “cargo” genes, some of which increase the fitness of the recipient host cell and can even make the hosts “addicted” to the respective MGE (Nicolas et al., 2015; Hülter et al., 2017; Benler et al., 2021). An obvious example includes horizontally transferred antibiotic resistance genes that become essential in the presence of antibiotics (Liebert et al., 1999). Antibiotic resistance or other cargo genes are determinants of MGE-host symbioses that in many cases are mutualistic. However, in this review, we focus on exaptations of genes directly involved in MGE mobility, replication or persistence as well as exaptation of entire MGEs.

## Main

### Exaptation of Mobile Genetic Elements and Their Components for Host Defense Functions

The potential for MGE to replicate at the expense of their hosts necessitates the evolution of systems that can discern self from non-self and protect the host from the deleterious effects of the parasites by curtailing their reproduction (Rimer et al., 2014). Defense systems can be partitioned into two discrete classes, based on whether they respond to fixed non-self patterns (innate immunity) or memorize variable non-self patterns and mount a response against specific parasites (adaptive immunity). The evolution of both types of immune systems and the parasites which they defend against are intrinsically entangled through exaptations (Koonin et al., 2017; Koonin and Makarova, 2017; Koonin et al., 2019)

The adaptive arm of prokaryotic immunity is effectuated by CRISPR-Cas, which is the evolutionary product of a constellation of mobile genetic elements. The memory function of CRISPR-Cas is achieved via Cas1 and Cas2, which jointly excise segments of nucleic acid from foreign genetic elements and insert the segments into the CRISPR repeat arrays in the host chromosome (Nuñez et al., 2014; Amitai and Sorek, 2016). Phylogenomic study of Cas1 uncovered ancestral homologs encoded by a distinct family of MGE, self-synthesizing transposons dubbed casposons (Krupovic et al., 2014; Krupovic et al., 2017). The evolutionary relationship between Cas1 and the homologous transposases of the casposons (dubbed ‘casposases’) is matched by extensive biochemical similarities between the two enzymes (Hickman and Dyda, 2015; Béguin et al., 2016; Hickman et al., 2020). Similarly, phylogenetic analysis of Cas2 demonstrates an evolutionary connection to mRNA-degrading toxins of the VapD family (Koonin and Makarova, 2013). The VapD ribonucleases are toxin components of toxin-antitoxin (TA) modules, which themselves exhibit properties of selfish elements and can be considered a type of MGE that typically piggy-back on plasmids (Jalasvuori and Koonin, 2015; Jurėnas et al., 2022). In the CRISPR adaptation complex, Cas2 performs a structural, scaffolding role, whereas the function of the nuclease activity, which is retained by most but not all Cas2 proteins, remain unknown (Amitai and Sorek, 2016; Sternberg et al., 2016). Thus, a transposase and a toxin apparently were the evolutionary grist for the memory capability of CRISPR-Cas, the defining feature of adaptive immunity.

Numerous type III CRISPR-Cas systems include a reverse transcriptase (RT) that is typically fused to Cas1 and enables adaptation by capturing spacers directly from RNA, either transcripts of DNA genomes of MGE, or possibly, RNA viruses (Koonin and Makarova, 2017; Silas et al., 2017). This CRISPR-associated RT is most closely related to the RT of prokaryotic retrotransposons (group II self-splicing introns), from which it was apparently recruited for the role in CRISPR adaptation (Koonin and Makarova, 2017; Silas et al., 2017). Thus, in these systems, the RNA-memorizing capability of CRISPR-Cas was endowed by the exaptation of a third mobile genetic element.

The effector complexes of CRISPR-Cas systems are highly diverse, apparently, owing to their capture from distinct mobile genetic elements. The architecture of the effector module distinguishes the two classes of CRISPR-Cas. The effector is either a multisubunit complex composed of several Cas proteins (class 1) or a single, large, multidomain protein (class 2) (Makarova et al., 2015; Makarova et al., 2020). The origins of class 1 effector modules remain murky. Nevertheless, comparative genomic analysis points to a likely ancestral relationship with a distinct variety of Abortive Infection (ABI) modules (Burroughs et al., 2015; Koonin and Makarova, 2019; Burroughs and Aravind, 2020). The ABI modules are a type of toxin-antitoxin systems that, after being activated by virus infection, induce cell dormancy or death, via a variety of mechanisms, of which the most common one is indiscriminate RNA cleavage, thus preventing virus reproduction and spread (Harms et al., 2018; Fraikin et al., 2020). Type III CRISPR-Cas systems possess the same functionality whereby the non-specific “altruistic” response is triggered by the specific target recognition. The inferred origin of type III effector modules from ABI systems implies that Class 1 CRISPR effectors started out as innate immunity systems that became the executive component of adaptive immunity though the merger with the adaptation module derived from casposons and TA.

The class 2 effectors have a completely different evolutionary history, being derived from nucleases encoded by MGE on multiple, independent occasions (Shmakov et al., 2015; Faure et al., 2019). One particular superfamily of transposable elements, the IS200/IS605, donated the nucleases (IscB and TnpB, respectively) that gave rise to Cas9 and Cas12, the effectors of type II and type V CRISPR-Cas systems (Kapitonov et al., 2016; Altae-Tran et al., 2021). The link between IscB and Cas9 is apparent from the shared, unique domain architecture of these proteins, in which an HNH nuclease domain is inserted with the RuvC-like nuclease. In contrast, Cas12 proteins only contain the RuvC-like nuclease domain, similarly to TnpB. The ancestral status of the transposon-encoded nucleases with respect to the CRISPR effectors is supported by general considerations, namely, the small size and compactness of IscB and TnpB, and the simple organization of the transposons themselves compared to the CRISPR-Cas systems (Koonin and Makarova, 2022). More importantly in the phylogenetic trees of the two nuclease families, Cas9 forms a single clade embedded amongst transposon-encoded IscBs (Altae-Tran et al., 2021), whereas different Cas12 variants comprise several such clades in the TnpB (Faure et al., 2019). Thus, while Cas9 was derived from a transposon-encoded nuclease IscB in a single evolutionary event (Kapitonov et al., 2016; Altae-Tran et al., 2021), Cas12 apparently evolved from transposon nucleases of the TnpB family on multiple, independent occasions (Faure et al., 2019; Altae-Tran et al., 2021). Similarly to Cas9 and Cas12, the nucleases of IS200/IS605-like transposons form a complex with a distinct guide RNA encoded within the same transposon (Altae-Tran et al., 2021). However, in these elements, the guide RNAs are not responsible for cleaving foreign DNA, but rather might direct the transposons to specific integration sites in the host chromosomal DNA; the details of the functions of these nucleases in transposons remain to be studied (Altae-Tran et al., 2021; Karvelis et al., 2021). Collectively, these observations indicate that most if not all major components of CRISPR-Cas systems originated via exaptation of MGE genes.

**FIGURE 1:**
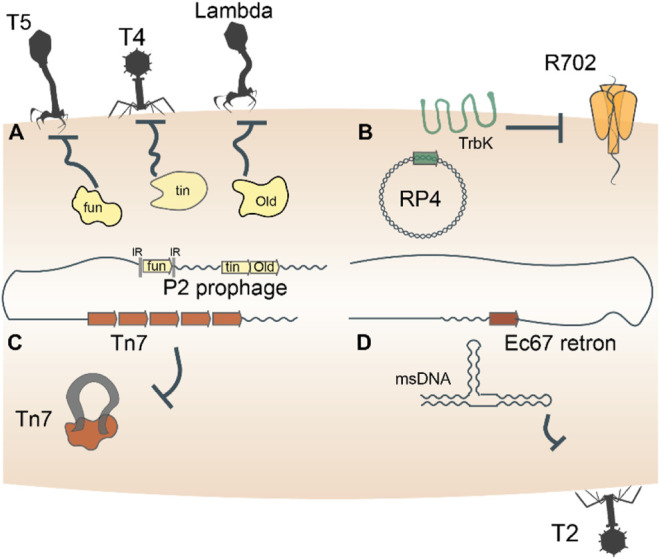
Entire mobile genetic elements double as defense systems. Autonomous MGEs, such as bacteriophages, plasmids, transposons and retrons, encode genes that defend the host cells against invasion by related or unrelated MGEs **(A–D)**. For example, *trbK* encoded by the broad host range plasmid RP4 excludes plasmid R702. Such defense-related genes are frequently shuttled back and forth between MGEs and their host cells, such as, for example, the Old family nuclease encoded by phage P2.

Prokaryotes harbor a multifarious armament of innate immune systems that defend against parasites, and many of these defense machineries were captured from MGE. In particular, bacteriophage (phage) superinfection exclusion systems serve as a rich depot from which prokaryotes can arm themselves for defense. Superinfection exclusion refers to the ability of a primary infecting phage to prevent a subsequent infection by another phage (Gentile et al., 2019; Owen et al., 2021), a form of inter-MGE competition. For example, phage P2 carries three genes, *fun*, *tin*, and *old*, which endow its host with immunity against phage T5, T-even phages, and lambdoid phages, respectively (Haggård-Ljungquist et al., 1989; Mosig et al., 1997; Odegrip et al., 2006) (Figure 1A). In both phage and bacterial genomes, the *fun* gene is flanked by inverted repeats that enable site-specific recombination and exchange of *fun* between bacteria and P2-like phages (Nilsson et al., 2004). Thus, through site-specific recombination, bacteria directly capture a superinfection exclusion gene from one phage that provides immunity to infection by other phages. Similarly, OLD is the archetypical member of a family of ABC-ATPases fused to a TOPRIM nuclease domain that is found in diverse defense contexts (Aravind et al., 1998; Krishnan et al., 2020). In combination with a UvrD-family helicase and RNAseH-family exonuclease, OLD is part of a widespread system that provides cells with innate immunity against several distinct bacteriophages (Doron et al., 2018; Cheng et al., 2021). The *old* and *fun* superinfection exclusion genes are just two examples of innate immune systems found in prokaryotic genomes associated with mobile genetic elements (Makarova et al., 2013), reflecting a broad evolutionary pattern of continuous back and forth gene shuffling.

Beyond the possession of genes dedicated to defense, mobile genetic elements themselves double as immune systems. As discussed above, bacteriophages furnish their hosts with immunity against secondary infections by related or unrelated phages through diverse mechanisms (Figure 1A). Plasmid incompatibility groups also can be viewed through the lens of inter-MGE competition, whereby the presence of one plasmid in a host cell precludes replication of another plasmid of the same incompatibility group (Cooper and Heinemann, 2000; Haase et al., 1996) (Figure 1B). Certain transposons including Tn7, Tn3 and Mu-like ones also exhibit defense phenotypes by rendering hundreds of kilobases of their genomic neighborhood refractory to integration of a second transposon (Arciszewska et al., 1989; Lambin et al., 2012; Stellwagen and Craig, 1997; Walker and Harshey, 2020) (Figure 1C). A distinct class of MGE, the reverse transcriptase-utilizing Retrons also abrogate bacteriophage infection of their hosts via mechanisms that remain to be characterized in detail (Gao et al., 2020; Millman et al., 2020) (Figure 1D). These “defensive” retrons might have been derived from an ancestral retrotransposon (group II intron). Generally, the dual role played by MGEs in both parasitizing and immunizing their host cells reflects the ‘shared interests’ between the two entities, which may be long-lasting or ephemeral.

### Recruitment of Mobile Genetic Elements Genes for Replication, Recombination and Repair

#### Chromosome Replication

Most prokaryotic chromosomes and some MGE replicons are covalently closed circular DNA molecules. Circular DNA poses a topological challenge for the proper segregation of genetic material upon cell division (Midonet et al., 2014). To face this challenge, MGEs and prokaryotes utilize resolvases to cleave dimers of covalently closed DNA molecules into monomers (Aravind et al., 2000). There is substantial evidence that prokaryotes repurposed tyrosine superfamily resolvases encoded by MGE for the faithful chromosome segregation.

**FIGURE 2:**
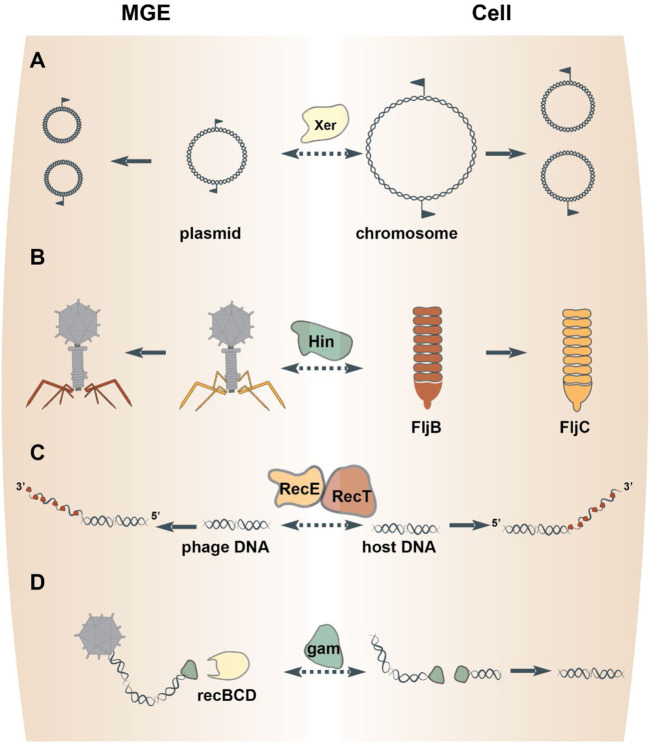
Proteins involved in DNA replication, recombination and repair freelance between MGEs and cells. Homologous resolvases mediate the resolution of both MGE and chromosome replication intermediates **(A)**. DNA inverting serine recombinases direct the expression of alternative phage tail fiber genes or host flagellum genes **(B)**. Exapted bacteriophage genes endow the host DNA repair pathways via homologous recombination **(C)** or nonhomologous end-joining **(D)**.

A well-characterized resolution system featuring a tyrosine superfamily resolvase is Xer/*dif.* Xer enzymes catalyze site-specific recombination at *dif* sites to resolve a circular DNA molecule into two individual molecules (Midonet et al., 2014) (Figure 2A). The bacterial XerC are closely related to the resolvases that mediate dimer resolution of plasmids and some Tn3-like transposons (Nicolas et al., 2015). Xer family recombinases are almost universal in bacteria and archaea (Smyshlyaev et al., 2021), but appear to have been displaced by homologs from MGEs on several occasions. In particular, chromosome segregation in *Streptococcus* and *Lactobacillus* is effectuated by XerS, a resolvase more closely related to those of bacteriophages than it is to XerC (Le Bourgeois et al., 2007; Cortez et al., 2010). In archaea, XerA resolves chromosomes, but can also recombine *dif* sites located on plasmids and exhibits signatures of recent acquisition from an integrated mobile element (Cortez et al., 2010; Midonet et al., 2014). Thus, prokaryotes routinely recruit MGE-encoded tyrosine resolvases to solve the topological problem of replicating a circular chromosome. Conversely, plasmids and phages can hijack bacterial Xer recombinases for the resolution of replication intermediates or, via the exaptation route, to integrate the phage genome into the bacterial chromosome (Midonet et al., 2014; Midonet et al., 2019).

MGEs also encode serine resolvases, which are unrelated to the tyrosine resolvases but play an analogous role (Aravind et al., 2000; Midonet et al., 2014; Smyshlyaev et al., 2021). To our knowledge, there is currently no evidence that prokaryotic chromosome segregation is ever mediated by serine resolvases, despite their analogous functionality.

Not all prokaryotes and MGE possess circular DNA replicons, some instead have linear molecules and covalently closed hairpin termini (Kobryn and Chaconas, 2002). Replication of such molecules also poses a topological challenge because bidirectional replication yields a circular dimer (Casjens, 1999; Kobryn et al., 2014). To face this challenge, MGE and prokaryotes encode telomerases that ensure the faithful segregation of linear DNA replicons. The paradigmatic telomerase is ResT, which is encoded by a linear plasmid in *Borrelia burgdorferi*. Indeed, loss of the plasmid-encoded ResT is lethal to *B. burgdorferi* due to defects in replicating the linear chromosomes (Byram et al., 2004). Thus, *B. burgdoreferi* outsources this critical step in DNA replication to an MGE.

#### Repurposed Mobile Genetic Element Enzymes Mediate Programmed Rearrangements of Host DNA and Proteins

Prokaryotes co-opted recombinases encoded by mobile genetic elements on multiple occasions to rearrange segments of chromosomal DNA. These recombinases belong to two unrelated superfamilies that are defined by the amino acid in their active site, either a serine or tyrosine. Chromosomal rearrangements mediated by enzymes of either superfamily serve to regulate transcription or create novel combinations of genes involved in various cellular processes (Weightman et al., 2002; Honarvar et al., 2003; Jiang et al., 2019).

One mechanism by which recombinases effectuate regulation is through DNA inversions, which either link alternative genes to the same promoter or switch the orientation of an individual gene’s promoter (Johnson, 2015). For example, DNA inversion mediated by the *Salmonella enterica* serine recombinase Hin results in the expression of one of two distinct flagellins (Johnson, 2015) (Figure 2B). Hin bears close sequence similarity to the serine recombinases encoded by the bacteriophages Mu and P1 (82% amino acid identity over the recombinase catalytic domain), which perform equivalent DNA inversion events in the phage genome to direct expression of different phage tail fibers (Harshey, 2015). Indeed, either phage enzyme can complement Hin-mutants to restore phase variation in *Salmonella enterica* (Simon et al., 1980). Such interchangeability of the DNA inverting serine recombinases between MGEs and their hosts has been documented for other pairs as well (Tominaga, 1997; Kutsukake et al., 2006). Thus, serine DNA invertases are frequently exchanged between MGEs and cells to regulate gene rearrangements.

A second mechanism by which recombinases effectuate regulation is through integration and excision of DNA. Enzymes from both serine and tyrosine recombinase superfamilies mediate the integration of all varieties of MGE, including plasmids, viruses and transposons, into prokaryotic chromosomes (Askora et al., 2011; Landy, 2015; Rice and Craig, 2015). In numerous cases where the MGE becomes incapable of horizontal transfer, such as via the loss of genes required for mobility, that MGE is repurposed as an excisable ‘switch’. For example, a defective prophage in *L. monocytogenes* is integrated into the *comK* gene, splitting the gene into two ORFs that are restored upon excision of the prophage (Pasechnek et al., 2020). Functionally analogous events mediate sporulation (Haraldsen and Sonenshein, 2003; Abe et al., 2017), nitrogen fixation (Golden et al., 1988; Carrasco et al., 1995) and DNA repair (Scott et al., 2008) in other prokaryotes. Thus, in many cases, integration and excision of MGEs catalyzed by their integrases serve to regulate transcription in prokaryotes.

Mobilization of prokaryotic self-splicing introns appears to provide a level of regulatory control to the benefit of the host cells, in which these elements reside. Mobile introns consist of a catalytic, self-splicing RNA (a ribozyme) and either a homing endonuclease (group I introns) or a RT-containing protein (group II introns) (Edgell et al., 2011). Although the mechanisms of mobility differ between group I and group II introns, ribozymes from both types of elements catalyze the intron excision from the parental RNA molecules (Hausner et al., 2014). Excision from parental RNA involves pairing of the extreme 5′ and 3′ ends of the intron at the intron-exon boundary and, consequently, restoration of the interrupted exon (Hausner et al., 2014). If the exon is a protein-coding gene, such restoration yields a fully functional protein upon translation. In *Clostridium difficile,* a group I intron is inserted upstream of a gene involved in bacterial virulence such that, upon excision, the ribosome-binding site is restored, stimulating translation (Lee et al., 2010). Critically, this group I intron lacks a homing endonuclease, and furthermore, the ribozyme activity is stimulated by a second messenger, cyclic diGMP, indicating that the ribozyme was exapted to tune translation (Lee et al., 2010). In other organisms, excision of group I or group II introns occurs in response to specific stimuli related to the gene product in which they reside (Belfort, 2017). For example, light stimulates excision of a group I intron from the photosynthesis gene *psbA* in *Chlamydomonas* chloroplasts (Deshpande et al., 1997; Lee and Herrin, 2003), suggesting that some introns function as molecular sensors of environmental cues.

Mobilization of a second, unrelated class of MGE, inteins, provides a degree of post-translational regulatory control. Like introns, inteins are mobilized to new DNA sites by homing endonucleases and are capable of self-splicing. Unlike introns, intein self-splicing and excision occurs after translation, from parental polypeptides (Novikova et al., 2016). Intein excision yields conditional post-translational regulatory control that is conceptually analogous to post-transcriptional control achieved by introns. For example, *Pyrococcus horikoshii* RadA hosts an intein that splices specifically in response to the presence of single-stranded DNA, the natural RadA substrate (Lennon et al., 2016). The RadA intein lacks a homing endonuclease and is therefore incapable of self transfer to new DNA sites, yet retains self-splicing capability (Lennon et al., 2016). Numerous other proteins host inteins that splice in response to environmental stimuli (Belfort, 2017), indicating that these inteins were repurposed as post-translational regulatory switches.

#### Capture of Mobile Genetic Elements Enzymes That Endow Prokaryotes With DNA Recombination and Repair Pathways

A cardinal mechanism of DNA repair is homologous recombination between paired strands of DNA. In *E. coli*, genetic analyses identified a homologous recombination pathway catalyzed by RecET (Kolodner et al., 1994). The nuclease activity of RecE generates single-stranded DNA overhangs that are subsequently bound by RecT to promote pairing and strand exchange between homologous segments of DNA (Kolodner et al., 1994) (Figure 2C). These enzymes are encoded by a defective prophage in *E. coli*, termed Rac (Kaiser and Murray, 1979). Rac is a prophage that lost ∼60% of its original DNA and is therefore incapable of replication or production of progeny virions (Casjens, 2003). Fully infectious Rac-like phages encode RecE homologs in similar genomic positions and mediate homologous recombination in their hosts (Figueroa-Bossi et al., 1997). This evidence indicates that the *E. coli* RecET homologous recombination pathway was captured from a temperate phage, perhaps, relatively recently because other, even closely related bacteria lack this prophage. Other bacteriophages encode ssDNA-binding proteins that promote homologous recombination, which belong to three unrelated superfamilies, RecT, Rad52 and ERF (Iyer et al., 2002). Like RecT, members from the other two superfamilies were captured from temperate phages because they are encoded sporadically throughout the bacterial domain and are flanked by phage-related genes that code for proteins involved in DNA recombination and repair (e.g., Holliday junction resolvases) (Iyer et al., 2002). Thus, genes of three unrelated superfamilies were recruited by bacteria from phages on multiple occasions for roles in homologous recombination.

A second mechanism of DNA repair is non-homologous end-joining (NHEJ). The joining reaction requires a DNA ligase and a DNA end-binding protein, known as Ku in eukaryotes. Homologs of Ku are present in prokaryotes (Aravind and Koonin, 2001; Doherty et al., 2001) and also mediate NHEJ by recruiting a DNA ligase and stimulating ligation of the two DNA ends (Weller et al., 2002). The prokaryotic Ku homologs are encoded within defective prophages related to the fully infectious *E. coli* phage Mu (di Fagagna et al., 2003). In Mu, the Ku homolog is known as *gam*, which is primarily involved in protecting Mu progeny from destruction by RecBCD during the lytic cycle, but can also mediate NHEJ of host DNA (Bhattacharyya et al., 2018) (Figure 2D). These observations collectively point to the shuttling of *gam* between phages and their bacterial hosts.

A special case of exaptation of MGEs to manipulate bacterial DNA is represented by Diversity-Generating Retroelements (DGR). DGRs are genetic cassettes composed of a reverse-transcriptase (RT) related to group II intron RTs, an accessory gene and *cis*-acting regulatory sequences (Doulatov et al., 2004; Handa et al., 2018). The DGRs broadly colonize both prokaryotic and phage genomes and introduce multiple mutations into specific target genes via highly error prone reverse transcription and retrohoming (Paul et al., 2017; Benler et al., 2018; Roux et al., 2021). The DGRs primarily introduce hypervariation into genes encoding cell-cell and virus-cell attachment but might contribute also to other cellular processes (Arambula et al., 2013; Vallota-Eastman et al., 2020). The frequent trafficking of DGRs between chromosomes, plasmids and phages (Wu et al., 2017) evinces the utility of accelerated protein sequence evolution mediated by this domesticated MGE.

### Co-Option of Transcription Factors From Mobile Genetic Elements for Host Cell Gene Regulation

**FIGURE 3:**
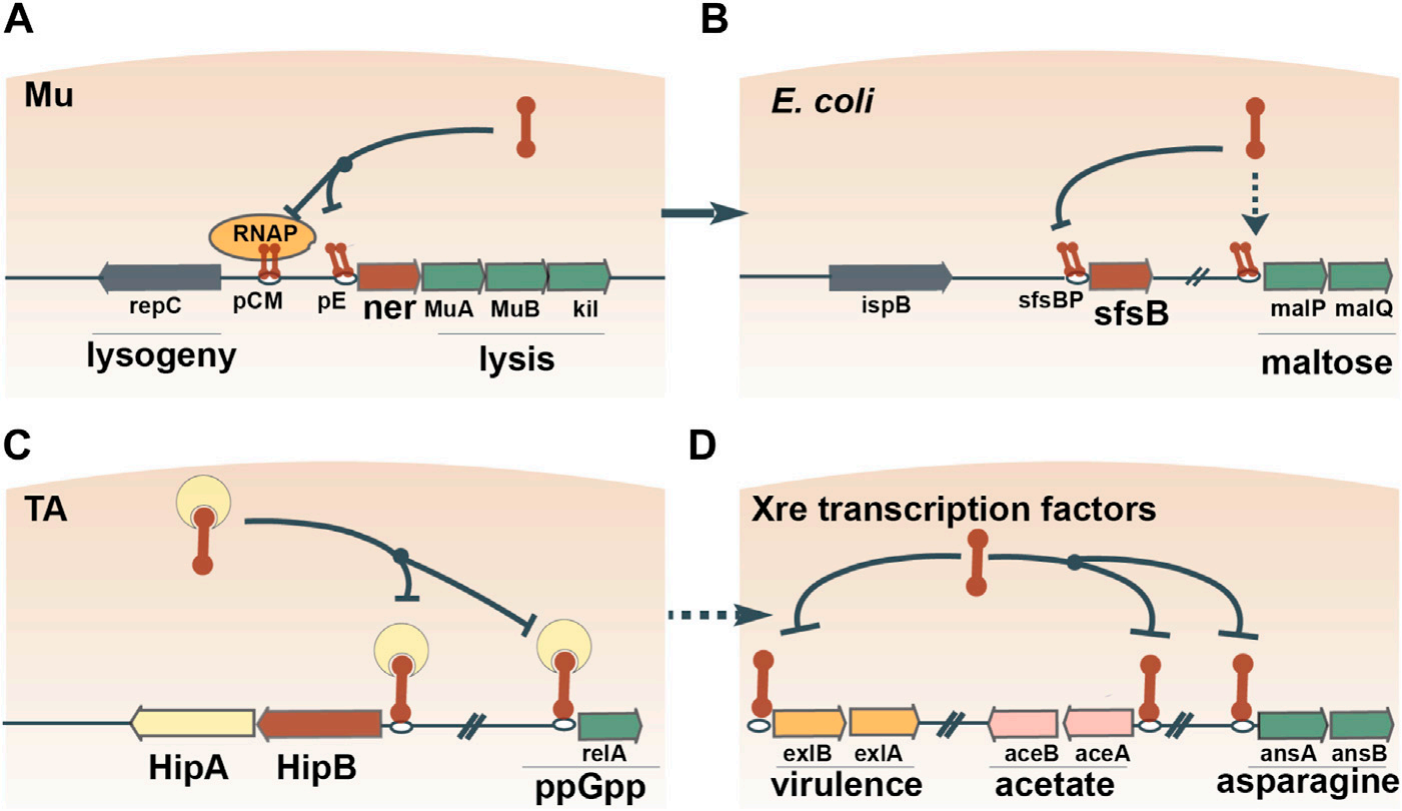
Recruitment of transcription factors from MGEs to regulate host gene expression. One example is Ner, which contains an Xre-family HTH domain and regulates the lysis-lysogeny switch of bacteriophage Mu **(A)**. The amino acid sequence of Ner is 68% identical to the maltose operon-activating transcription factor SfsB, indicating the recent recruitment of a Ner homolog for the regulation of host carbon metabolism **(B)**. Xre domains are widespread in antitoxin proteins, such as HipB, which autoregulate their own expression and the expression of other host genes **(C)**. Antitoxins often disassociate from their TA operons and assume dedicated roles as transcription factors **(D)**.

On multiple occasions, prokaryotes seem to have adopted MGE-encoded transcription factors to regulate transcriptional networks. The most common DNA-binding moiety in prokaryotic transcription factors is the ubiquitous helix-turn-helix (HTH) domain (Brown et al., 2003; Cuthbertson and Nodwell, 2013; Hoskisson and Rigali, 2009). Due to the small size of the HTH domain, robust phylogenetic reconstruction of the evolutionary history of HTH domain-containing genes proves difficult. Nevertheless, apparent monophyletic groups can be identified, several of which show clear signs of exchange between prokaryotes and MGEs (Aravind et al., 2005). The principal signature of such an event is the widespread presence of a given family of HTH domain-containing proteins in MGEs and a restricted distribution in prokaryotes. For example, Xre is the archetypical member of one of the families of HTH domains with tetrahelical quaternary structure (Aravind et al., 2005). Xre regulates the lysis-lysogeny of a degraded prophage in *B. subtilis* (McDonnell and McConnell, 1994), and homologs of Xre regulate the lysis-lysogeny decisions of autonomous bacteriophages, for example, the *ner* gene encoded by *E. coli* phage Mu (Tolias and Dubow, 1986) (Figure 3A). Ner exhibits 68% amino acid identity to SfsB (NP_417655.1), a transcription factor that is conserved in Enterobacteriaceae and regulates genes involved in maltose metabolism (Autexier and Dubow, 1992) (Figure 3B). The high sequence similarity between Ner and SfsB suggests a relatively recent exchange. Furthermore, overexpression of *ner* can complement *sfsB* by stimulating the expression of maltose metabolic genes in *E. coli* (Autexier and Dubow, 1992), underscoring the ease with which a transcription factor from a MGE can be recruited into the host regulatory cascades.

The antitoxins of toxin-antitoxin (TA) pairs have also been shown to double as cellular transcription factors, illustrating another plausible route of exaptation. Specifically, Xre-family HTH domains are widespread in antitoxins of type II TA systems (Freire et al., 2019; Makarova et al., 2009; Skjerning et al., 2019). Typically, the TA complex autoregulates its own transcription by binding to an operator(s) upstream of the TA operon via the HTH domain (Page and Peti, 2016). In *E. coli,* two different antitoxins bind to the operators of genes located outside of the cognate TA pair and simultaneously regulate multiple operons (Lin et al., 2013; Soo and Wood, 2013) (Figure 3C). These observations suggest that antitoxins first assume regulatory control of host genes, and then, disassociate from the TA pair and become dedicated transcription factors. Transcription factors containing Xre-family HTH domains regulate operons involved in various processes, such as virulence (Trouillon et al., 2020), acetate metabolism (Gerstmeir et al., 2004), asparagine metabolism (Sun and Setlow, 1993) and propionyl coenzyme A assimilation (Carter and Alber, 2015) (Figure 3D). Overall, Xre homologs function as transcription factors that often switch between regulatory roles in MGE and host gene expression.

The incorporation of MGE-encoded transcription factors into host regulatory networks likely extends beyond the Xre-family of HTH domains. For example, Ribbon-Helix-Helix (RHH) domains are common in MetJ/Arc-family transcription factors as well as in TAs, suggesting that the bacterial transcription factors of this family were originally derived from antitoxins (Aravind et al., 2005). Given that TAs and other MGEs are activated by various signals, such as DNA damage (Knowles et al., 2017) or the presence of other MGE (McKitterick and Seed, 2018; LeGault et al., 2021), recruitment of MGE-encoded transcription factors could be favorable for the host, enabling it to respond to the same stressors (Benler and Koonin, 2020).

### Exaptation of Mobile Genetic Elements Genes for Functions in Cell Cycle Control, Cell Division, Chromosome Partitioning

Both MGEs and prokaryotic cells employ partitioning systems that ensure inheritance of DNA by the daughter cells upon binary fission of a parental cell. Three well-characterized partitioning systems all require an NTPase, a centromere-like site and a DNA-binding adaptor protein that connects the two (Gerdes et al., 2010). The paradigmatic ParABS system contains a P-loop superfamily ATPase (ParA) and was originally characterized for its role in plasmid segregation (Ogura and Hiraga, 1983). ParA can also orchestrate the segregation of the chromosome on which they reside (Gerdes et al., 2010). Phylogenetic analysis largely separates plasmid and chromosomal *parA* genes, but in some cases, ParA genes of plasmid origin are encoded on chromosomes (Gerdes et al., 2000) and are necessary for their segregation (Yamaichi et al., 2007). Furthermore, even within the plasmid-dominated branch of ParA homologs, a subgroup exists that is represented by the cell division proteins MinD and Mrp of *E. coli* (Gerdes et al., 2000). Therefore, the parsimonious evolutionary scenario appears to involve exaptation of ParA from an MGE for the function in bacterial chromosomal DNA segregation and cell division.

Beyond the capture of MGE-encoded partitioning enzymes, the chromosomes of some prokaryotes themselves might originate from MGEs. One prominent example is the second chromosome of *Vibrio cholerae*, which is hypothesized to derive from an ancestral plasmid (Heidelberg et al., 2000), given the presence of multiple TA systems that are common addiction modules carried by plasmids and other MGEs (Makarova et al., 2009). Furthermore, as discussed above, the Par genes encoded by chromosome II of *V. cholerae* are phylogenetically closely related to plasmid Par genes, in contrast to those encoded on chromosome I that appear to be genuine cellular genes (Gerdes et al., 2000). Deletion of the *par* genes on chromosome II results in its loss upon cell division and is followed by cell death, which phenotypically resembles programmed cell death caused by free toxins released from their cognate antitoxins (Yamaichi et al., 2007). Together, these observations suggest that *V. cholerae* chromosome II evolved from a TA-carrying plasmid the maintenance of which was further reinforced by the capture of essential, housekeeping genes (McGeoch and Bell, 2008; Hülter et al., 2017). In other bacteria with multiple chromosomes, the ParS sites differ substantially between the primary and secondary chromosomes, again suggesting distinct evolutionary histories, with the secondary chromosomes evolving from plasmids (Livny et al., 2007). Thus, conversion of plasmids into chromosomes could be a common route of evolution in prokaryotes and seems to represent a distinct form of exaptation that involves “domestication” of an MGE replicon itself.

### Mobile Genetic Elements Repurposed for Intra- and Intercellular Trafficking, Secretion and Vesicular Transport

**FIGURE 4:**
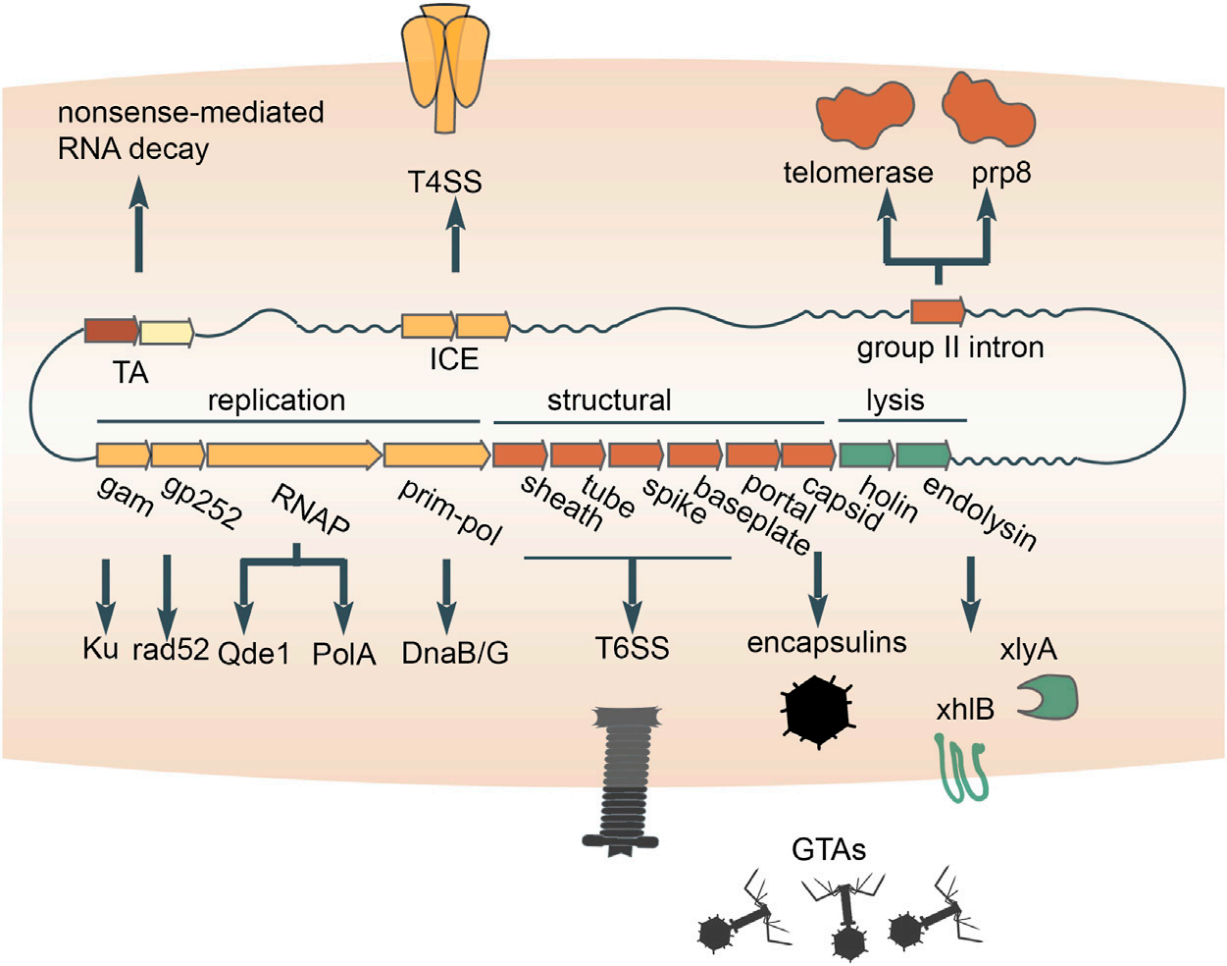
Multiple contributions of distinct mobile genetic elements to intercellular transfer pathways and eukaryogenesis. Exaptations of toxin-antitoxins (TAs), integrative and conjugative elements (ICE), bacterial retrotransposons and double-stranded DNA bacteriophages are diagrammed. An RNA-cleaving toxin apparently was incorporated into the eukaryotic nonsense-mediated RNA decay system. The conjugation apparatus of ICE was exapted for the transfer of proteins (type 4 secretion systems). Recruitment of the reverse transcriptase from bacterial retrotransposons yielded the key component of the eukaryotic spliceosome, Prp8 (accompanied by inactivation of the reverse transcriptase), as well as telomerases which retain the activity. The structural module of double-stranded DNA bacteriophages was repurposed for the delivery of proteins (type 6 secretion systems) or host DNA (GTAs) between cells. The replication and lysis modules donated multiple genes that play diverse roles in both prokaryotes and eukaryotes.

Bacteriophage tails have been domesticated on multiple independent occasions for the secretion and transfer of gene products from bacterial cells and/or transfer to other cells. In particular, some *Uroviricota* phages (e.g., T4) possess contractile tails that puncture their host cell envelopes and serve as a conduit for the delivery of encapsidated DNA and proteins into the cytoplasm (Miller et al., 2003). Phage tails were neofunctionalized to secrete proteins to the benefit of the cell (Leiman et al., 2009; Lossi et al., 2013; Pell et al., 2009). Such devices, known as type VI secretion systems, are widespread and were likely captured from different phages on independent occasions (Böck et al., 2017; Denise et al., 2020) (Figure 4). The proteins transferred by type VI secretion systems are commensurately diverse. but many function as toxins that exert anti-bacterial or anti-eukaryotic activity (Cherrak et al., 2019). One such toxin apparently evolved from the phage tail tip protein (M. Iyer et al., 2021), whereas numerous other T6SS toxins possess cognate antitoxins (Nolan et al., 2021), suggesting that TA systems were recruited for inter-species competition. Other proteins secreted via phage tails perform non-competitive roles (Russell et al., 2014). For example, a phage tail-like structure produced by *Pseudomonas luteoviolacea* delivers a cargo protein that stimulates the metamorphic transition of marine tubeworm larvae into juveniles (Ericson et al., 2019; Cavalcanti et al., 2020). Overall, domesticated bacteriophage tails represent a major route for the secretion of diverse proteins from prokaryotic cells and their delivery to various targets.

Prokaryotes transfer proteins and DNA across cell envelopes via an apparatus that was captured from conjugative mobile genetic elements. The MGEs that self-transfer via conjugation include plasmids and transposons. One of the key enzymes involved in conjugation is an HUH-superfamily endonuclease (Ilyina and Koonin, 1992), which nicks a DNA strand of the MGE prior to transfer (Alvarez-Martinez and Christie, 2009). A second, conserved enzyme is an FtsK/HerA-superfamily ATPase that pumps DNA bound to the endonuclease through mating bridges (Iyer et al., 2004). Phylogenomic analysis of the conserved ATPase shows that conjugation systems of MGEs repeatedly lose the endonuclease and thus can no longer mediate self-transfer (Guglielmini et al., 2012). These non-autonomous MGEs mediate non-conjugation-related secretion of protein and DNA (Guglielmini et al., 2012). By coopting the conjugative machinery from MGEs (Figure 4), prokaryotes opened up another route for the transfer of macromolecules out of the cell.

Another route by which prokaryotes transfer DNA is through repurposed bacteriophage virions. Highly degraded and fragmented phage genomes integrated in the genomes of many bacteria package host DNA, rather than the prophage DNA, into mini-phage virions (Bárdy et al., 2020; Esterman et al., 2021; Kogay et al., 2019; Lang et al., 2012; Shakya et al., 2017) (Figure 4). Because these mini-phage particles lyse the cell from within and transfer the packaged DNA to a recipient host, they are known as Gene Transfer Agents (GTA), where the transfer of DNA from the primary host cell is thought to confer a selective advantage for the recipient cells and ultimately for the population as a whole (Lang et al., 2012). Thus, the GTAs are domesticated, defective phages that have been exapted to serve as dedicated vehicles for DNA transfer within microbial populations. A notable, related system are the pirate phages, such as *Staphylococcus aureus* pathogenicity islands (SaPis). The SaPis and other pirate phages spread by hijacking the particles of a superinfecting phage (Novick and Ram, 2017; Novick, 2019). However, the SaPi particles are harmless to the host bacterium because they have no capacity to reproduce via the lytic cycle. In this process, the pirate phage both spreads its genome and protects the host population from killing by the pirated phage.

Besides mediating intercellular transfer of macromolecules, capsids of dsDNA phages might have been enlisted for the intracellular trafficking and compartmentalization of proteins. All tailed dsDNA bacteriophages and archaeal viruses (virus realm Duplodnaviria) encase their genomes within icosahedral capsid made of the HK97-fold major capsid protein (Koonin et al., 2020). Shells built from HK97-fold proteins with significant similarity to phage capsid proteins are encoded by standalone genes in numerous bacterial and archaeal genomes, where they form icosahedral particles known as encapsulins, which sequester diverse cargo proteins (Fontana et al., 2014; Giessen and Silver, 2017; Twarock and Luque, 2019; Nichols et al., 2021). Although the specific evolutionary relationships between encapsulins and phage capsids remain to be elucidated, a plausible evolutionary scenario is that encapsulins were domesticated from double-stranded DNA viruses on one or more occasions (Krupovic and Koonin, 2017) (Figure 4), providing prokaryotes with a means to sequester reactants into a nanocompartment.

Genes captured from bacteriophages contribute to the formation of bacterial biofilms, vesicles and spores. The proteins encoded in the phage lysis gene cassettes permeabilize cytoplasmic membranes and enzymatically degrade host cell peptidoglycan from within, releasing progeny viral particles for subsequent infections (Cahill and Young, 2019). Programmed lysis by phage lytic genes releases the macromolecular components of the cell, in particular DNA, a principal constituent of biofilm matrices. Biofilm matrix formation mediated by the lysis cassette of a domesticated bacteriophage has been observed in *Pseudomonas aeruginosa* (Heussler et al., 2015). In *Bacillus subtilis*, phage lysis cassettes instead mediate the formation of membrane vesicles (Toyofuku et al., 2017) or spores (Real et al., 2005). In Caulobacterales*,* a phage lytic enzyme was coopted as a key gene required for cellular morphological development (Randich et al., 2019). These examples highlight the utility of phage lysis cassettes for cellular wall remodeling or destruction of individual cells within larger populations, resulting in population level benefits.

### Multiple Contributions of Prokaryotic Mobile Genetic Elements to Eukaryogenesis

Apart from their diverse input to the evolution of various functional systems in bacteria and archaea, prokaryotic MGE made major contributions to the origin of eukaryotes, partly, through the mitochondrial endosymbiont. Here we can give only a brief account of these recruitments of MGE genes, but leaving them out would fail to give justice to the evolutionary role of these MGE. Most bacteriophages encode polymerases that replicate and transcribe their genetic information, and on at least one occasion, such polymerases displaced the functionally analogous bacterial polymerases during eukaryogenesis. Strikingly, three enzymes that are encoded in eukaryotic nuclear genomes and involved in the replication and transcription of mitochondrial genomes have readily traceable phage ancestry. Specifically, the mitochondrial DNA-dependent DNA polymerase (DNAP) of the A family, DnaB-DnaG-like helicase-primase and single-subunit DNA-directed RNA polymerase (RNAP) are all more closely related to the corresponding polymerases of T7/T3-like phages than to any bacterial polymerases (Filée et al., 2002) (Figure 4). The case of the RNAP is particularly notable because the phage single subunit RNAP, originally apparently derived from a bacterial A family DNAP (Cheetham and Steitz, 2000), became the enzyme responsible for the expression of the mitochondrial genome, displacing the multisubunit RNAP that is universal in all cellular life forms. Most likely, all these enzymes were exapted from a prophage that was integrated in the genome of the ancestral α-proteobacterium that gave rise to mitochondria as a result of endosymbiosis (Filée and Forterre, 2005; Shutt and Gray, 2006). The non-orthologous displacement of the RNAP occurred early in the evolution of the mitochondria, but probably, many millions of years post-endosymbiosis because the mitochondrial genomes of at least some jacobids, such as *Reclinomonas americana*, encode a typical bacterial multisubunit RNAP (Burger et al., 2013; Gray et al., 2020).

Many components of eukaryotic innate immune and damage control systems as well as repair and splicing machineries seem to derive from prokaryotic MGEs. Such connections include the apparent origin of components of the eukaryotic nonsense-mediated mRNA decay system from bacterial TA modules (Anantharaman and Aravind, 2003) and the animal apoptosis proteins Bax/Bak from phage lysis cassettes (Saier et al., 2015). A component of the eukaryotic RNAi machinery, the QDE1 family RNA-dependent RNA polymerase, appears to have evolved from a distinct bacteriophage RNAP (Drobysheva et al., 2021; Iyer et al., 2003; Shabalina and Koonin, 2008) (Figure 4). Two proteins that play central roles in homologous recombination and NHEJ (double-strand break repair) in eukaryotes, Rad52 and Ku, respectively, appear to have originated in bacteriophages (Aravind and Koonin, 2001; di Fagagna et al., 2003). Both the telomerase that restores chromosomal termini and the key protein of the eukaryotic spliceosome were captured from a reverse-transcriptase encoded by group II introns, that is, bacterial retrotransposons (Dlakić and Mushegian, 2011; Gladyshev and Arkhipova, 2011; Lambowitz et al., 2015) (Figure 4). The relationships between eukaryotic proteins and their ancestors from prokaryotic MGE are often subtle and hard to detect, so the full extent of the contribution of these elements to eukaryogenesis awaits a systematic investigation with the most powerful available tools for protein sequence and structure comparison.

## Concluding Remarks

The broad repertoire of exaptations surveyed here extends to numerous functional systems, abundantly illustrating the evolutionary entanglement between MGEs and their prokaryote hosts. Nevertheless, the overarching principle inferred from the study of defensive exaptations is readily applicable. That is, molecular components evolved by MGEs are expediently recruited for mechanistically similar but biologically distinct roles in the cell owing to their fundamental biochemical utility. This principle has been captured in the “guns for hire” metaphor (Koonin et al., 2019), which emphasizes the perennial shuttling of genes, gene modules and whole replicons between MGE and their hosts. The most prominent contribution of prokaryotic MGE is to the molecular componentry of functional systems that are involved in various biological conflicts, in particular, defense against viruses and other MGEs. To wit, the complex molecular machinery of CRISPR, the prokaryotic adaptive immune system, apparently was assembled primarily if not completely from components exapted from MGE. Moreover, restriction-modification and TA modules, the most common innate immunity systems in prokaryotes, themselves can be considered a distinct variety of MGE that, while lacking their own replicative machinery, attain extensive horizontal mobility by routinely piggy-backing on plasmids and viruses (Kobayashi, 2001; Fraikin et al., 2020). This evolutionary entanglement of MGE and defense systems appears to be far from accidental but rather reflects a deep unifying feature. Indeed, both types of genetic elements are generally deleterious, stronger in the case of MGE and weaker in the case of defense systems (that are beneficial only during the brief periods of exposure to the respective MGEs), to the organisms in which they reside (Iranzo et al., 2017). Therefore, these elements evolved and exploit various mechanisms of survival that include horizontal mobility, causing host addiction, and exaptation for roles beneficial to the host.

It has been noted that replicators form a continuous spectrum with regard to the degree of their replicative autonomy and cooperativity (Jalasvuori and Koonin, 2015; Koonin et al., 2019). The numerous cases of exaptation and shuttling of components between MGE and hosts as well as among different varieties of MGE show that this continuity also encompasses uninterrupted flow of genetic material across the spectrum. Crucially, the coevolution of MGEs and their cellular hosts cannot be reduced to arms race, but rather involves the entire gamut of cooperation, inter-MGE competition, and exaptation. Exaptation of MGE and their components pervades the history of most if not all cellular organisms, and hardly any MGE seem to evade exaptation of at least some of their components. Moreover, these exaptations substantially contributed to evolutionary transitions, such as the origin of eukaryotes. Comprehensive investigation of the flow of genetic information between MGE and cellular life forms should provide major insight into the evolution of life.
